# Hunting strategies used in the semi-arid region of northeastern Brazil

**DOI:** 10.1186/1746-4269-5-12

**Published:** 2009-04-22

**Authors:** Rômulo RN Alves, Lívia ET Mendonça, Maine VA Confessor, Washington LS Vieira, Luiz CS Lopez

**Affiliations:** 1 Departamento de Biologia, Universidade Estadual da Paraíba, Av. das Baraúnas, 351/Campus Universitário, Bodocongó, 58109-753, Campina Grande, Paraíba, Brasil; 2 Programa de Pós-Graduação em Ciências Biológicas (Zoologia), Laboratório e Coleção de Herpetologia, Departamento de Sistemática e Ecologia, Universidade Federal da Paraíba, 58051-900, João Pessoa, PB, Brasil; 3 Departamento de Sistemática e Ecologia, Universidade Federal da Paraíba, 58051-900 João Pessoa, PB, Brasil

## Abstract

Hunting for wild animals is stimulated by the many different human uses of faunal resources, and these animals constitute important subsistence items in local communities in the Caatinga region. In order to gain access to these resources, hunters have developed a series of techniques and strategies that are described in the present work. The principal hunting techniques encountered were: waiting, especially directed towards hunting diurnal birds; calling ("arremedo"), a technique in which the hunters imitate the animal's call to attract it to close range; hunting with dogs, a technique mostly used for capturing mammals; tracking, a technique used by only a few hunters who can recognize and follow animal tracks; and "facheado", in which the hunters go out at night with lanterns to catch birds in their nests. Additionally, many animal species are captured using mechanical traps. The types of traps used by the interviewees were: dead-fall traps ("quixó"), iron-jaw snap traps ("arataca"), wooden cages with bait ("arapuca"), iron-cage traps ("gaiola'), "visgo", multi-compartment bird cages ("alçapão"), buried ground traps with pivoted tops ("fojo"), and nooses and cages for carnivorous. The choice of which technique to use depends on the habits of the species being hunted, indicating that the hunters possess a wide knowledge of the biology of these animals. From a conservation perspective, active hunting techniques (waiting, imitation, hunting with dogs, and "facheado") have the greatest impact on the local fauna. The use of firearm and dogs brought greater efficiency to hunting activities. Additional studies concerning these hunting activities will be useful to contribute to proposals for management plans regulating hunting in the region – with the objective of attaining sustainable use of faunal resources of great importance to the local human communities.

## Background

The relationships between men and animal species represent some of the most ancient types of human interactions with the biodiversity of our planet. As Holland [1] pointed out, pre-historic societies used animals and their products (primarily consumed as food), and the use of animals has perpetuated throughout the history of humanity. Wild animals and their body parts or sub-products are used in a wide variety of ways in contemporary societies: as food resources, as pets, in cultural activities, for medicinal and magic-religious purposes, as clothing and tools [2-11]. The many uses of faunal resources have always stimulated hunting – one of the most ancient human activities – which continues, to a greater or lesser extent, to the present day [12-17].

Animals have been used for numerous purposes by indigenous societies for millennia in Brazil and by Europeans since colonial times. The country retains between 15 and 20% of the world's biodiversity – as well as a huge cultural diversity represented by more than 200 indigenous tribes and a large number of traditional communities that all possess considerable knowledge about the local fauna and flora and use these natural resources in many different ways [18]. Little attention, however, has been given to this social use of the biodiversity in Brazil and the few works that have been published on the subject were studies undertaken in the Atlantic and the Amazonian Forests [19-21]. There have been no published studies about subsistence hunting for the Caatinga Biome, even though it is known to be one of the greatest threats to the regional faunal biodiversity [22] and numerous animal species there are threatened by extinction due to intense hunting pressure and environmental degradation [23,24]. From a social perspective, on the other hand, the capture of wild animals constitutes an important factor in the subsistence of the human communities inhabiting Brazil's semi-arid northeastern region.

In the face of the need to develop environmental conservation strategies suited to the socioeconomic and ecological realities of human extractive activities in the Caatinga, the present study describes techniques and strategies used there for hunting animals. Our results should be useful in formulating management plans and proposals for regulating hunting while permitting sustainable use of the faunal resources of that region.

## Materials and methods

### Study area

**Figure 1:**
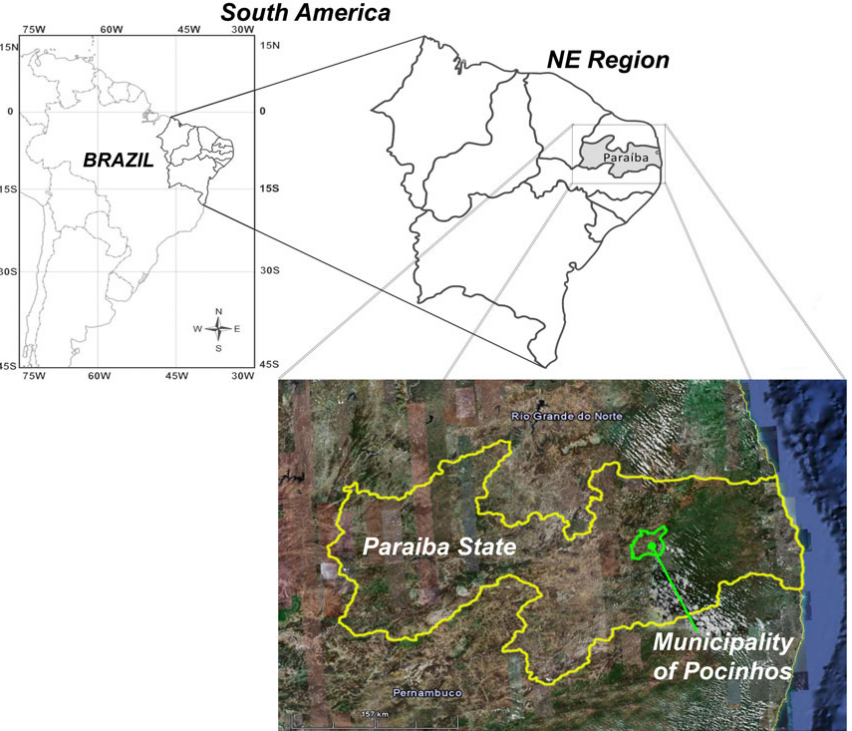
**Map of study area, Municipality of Pocinhos, NE Brazil**.

The present study was carried out in the municipality of Pocinhos, located in the western sector of the Borborema Plateau, Paraíba State, Pocinhos micro-region, Paraibano meso-region, Brazil (Fig. 1) [25]. The municipality of Pocinhos is bordered by Campina Grande, Boa Vista, Puxinanã, Soledade, Olivedos, Barra de Santa Rosa, Algodão de Jandaíra, Esperança, Areial, and Montadas [26]. Pocinhos occupies an area of 630 km^2 ^and has a population of approximately 14 880, of which about fifty percent (7,323) reside in the rural zone [27].

The average annual temperature is 23°C and varies little during the year. The region has a very low rainfall rate, oscillating annually between 400 and 600 mm. The climate is hot, semi-arid, with rainfall in the autumn and winter months (dry Mediterranean type) [26] and the vegetation is dominated by sub-deciduous and deciduous forests typical of semi-arid regions. Soil quality is very variable, with a certain predominance of average to high fertility areas [25]. The regional topography presents a rolling landscape dissected by deep, narrow valleys.

### Procedures

Information concerning hunting practices and strategies was obtained through semi-structured questionnaires complemented by free interviews [28]. The questionnaires were applied to 106 hunters from the municipality and, of this number, 78 (74%) live in urban areas but frequently travel to rural areas to hunt, while 28 (26%) live in the rural zone. Among the interviewees, key-informants (more experienced hunters) were selected using the criterion of "native specialists" – people who consider themselves, and are considered by the community, as culturally competent in this area [29,30]. We accompanied the 15 informants (8 rural and 7 urban) in their hunting activities to acquire better descriptions of the hunting techniques used for the most important game species. Demographics of the interviewees are summarized in Table 1.

**Table 1:** Information on educational attainment, age, income, and gender of interviewees.

Gender	
Male	104 (98.12%)
Female	2 (1.88%)
Age	
29 or younger	29 (27.9)
30–39	20 (18.6)
40–49	10 (9.3)
50–59	18 (17.44)
60–69	15 (13.95)
70 or older	14 (12.79)
Monthly income*	
Undeclared	25 (23.58)
Less than minimum wage	26 (24.41)
One to two times minimum wage	42 (39.53)
Two to three times minimum wage	7 (6.97)
Three to four times minimum wage	4 (3.48)
Four to five times minimum wage	2 (2.32)
Educational attainment	
Illiterate	53 (50)
Attended school for 8 years	40 (38.36)
Attended school for less than 8 years	4 (3.48)
Finished high school	9 (8.13)

* Brazilian minimum wage approximately equivalent to US$ 180 at the time surveys took place.

During the interviews the hunters answered questions about each of the animals they hunted, their hunting techniques, and reasons for hunting these animals, etc. Prior informed consent was obtained for all interviews conducted. The ethical approval for the study was obtained from the Ethics committee of Paraiba University State.

The animals cited as being hunted were identified by the analysis of specimens captured and donated to the project, photographs of the animals taken during the interviews, and based on their vernacular names (with the aid of taxonomists familiar with the fauna in the study area).

## Results

Hunting in the *Caatinga *region has been practiced since remote times and represents a traditional form of wildlife management. Hunting in the study area is associated with the following categories: a) subsistence (food resources) (n = 20 hunters); b) control (directed towards animal predators considered dangerous to humans or their domestic animals or to the dogs used for hunting) (n = 4); c) sport (leisure and entertainment) (n = 82). A total de 56 hunters who were interviewed indicated that they hunted for two or more motives, and one of them was hunting to control predators. In addition to the animals being used as food, these creatures could also be kept as pets, used for medicinal and magic-religious purposes, or as clothing or for tools.

**Figure 2:**
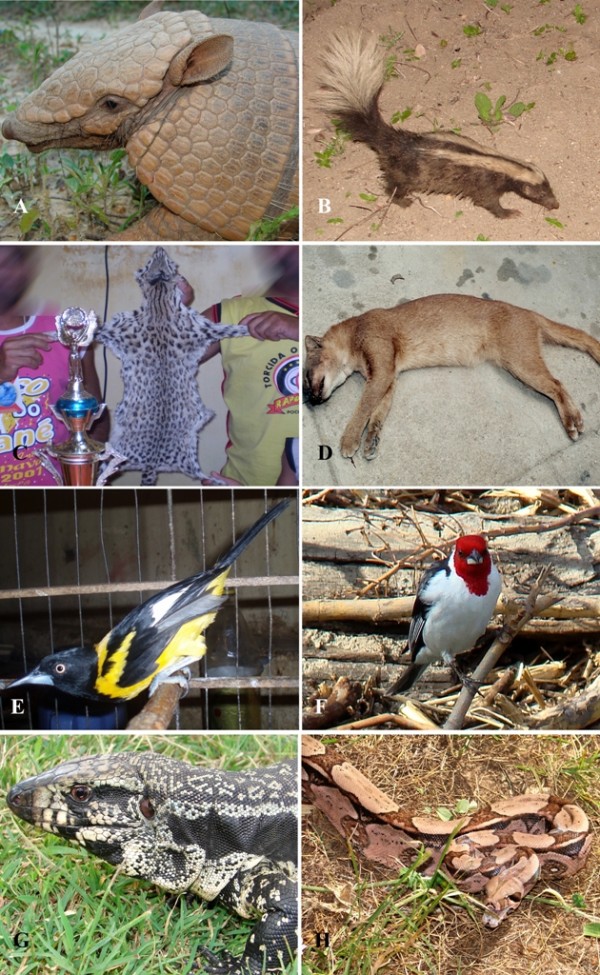
**Examples of animals hunted in the semi-arid northeastern region of Brazil**. A: *Euphractus sexcinctus*, B: *Conepatus semistriatus*, C: Skin of *Leopardus tigrinus*, D: *Puma yagouaroundi*, E: *Icterus jamacaii*, F: *Paroaria dominicana, G: Tupinambis merianae *and H: *Boa constrictor *(Photos – A, B, G, H: Washington Vieira; C, D, E: Lívia Mendonça and F: Hélder Araújo).

The most hunted prey were: *Kerodon rupestris *(rock cavy – "mocó"), *Conepatus semistriatus *(striped hog-nosed skunk – " tacaca"), *Dasypus novemcinctus *(nine-banded armadillo – "tatu verdadeiro"), *Euphractus sexcinctus *(six-banded armadillo – "tatu peba"), *Tamandua tetradactyla *(southern tamandua – "tamanduá"), *Leopardus tigrinus *(small spotted cat – "gato maracajá"), *Puma yagouaroundi *(jaguarundi – "gato vermelho"), *Cerdocyon thous *(crab-eating fox – " raposa"), *Galea spixii *(spix's yellow-toothed cavy – "preá"), *Cavia aperea *(Brazilian guinea pig – "preá"), *Galictis vittata *(greater grison – "furão"), *Didelphis albiventris *(skunk – 'timbu'), *Zenaida auriculata *(eared Dove – "ribacã"), *Claravis pretiosa *(blue ground-dove – "rolinha"), *Columbina picui *(picui ground dove-" rolinha"), *C. minuta *(plain-breasted ground-dove – "rolinha"), *C. squammata *(scaled dove-" rolinha"), *C. talpacoti *(ruddy ground-dove – "rolinha"), *Nothura maculosa *(spotted nothura – "cordoniz"), *Leptotila verreauxi *(white-tipped dove – "jurutis"), *Crypturellus tataupa *(tataupa tinamou – " lambus"), *C. parvirostris *(small-billed tinamou – "lambus"), *Cariama cristata *(red-legged seriema), *Patagioenas picazuro *(picazuro pigeon – "asa branca"), *Aramides *sp (wood rail – "sericóia"), *Icterus jamacaii *(campo troupial – "concriz"), *Cyanocompsa brissonii *(ultramarine grosbeak – "azulão"), *Carduelis yarrellii *(yellow-faced siskin – "pinta silva"), *Sporophila albogularis *(white-throated seedeater – "golado"), *Gallinula chloropus *(common moorhen – galinha dágua), *Paroaria dominicana *(red-cowled cardinal – "galo de campina"), *Tupinambis merianae *(lizard, "tegu" – "tejuaçú"), *Crotalus durissus *(rattlesnake – "cascavel"), *Boa constrictor *(boa – "jibóia"), *Micrurus *sp. (coral snakes) and *Bothrops *sp. (vipers). Examples of hunted animals are shown in Figure 2.

Hunting knowledge is passed from generation to generation and is part of the culture of the people who live in the *Caatinga *region. Hunting activities start in early childhood when animals (generally birds and reptiles) are hunted for food using "baladeiras" (sling-shots), or captured in traps (generally birds) and turned into pets. Hunting among adults is carried out using various capture techniques that are adapted to the type of prey and the habitat where the species live. The techniques and strategies used by hunters are described below:

### Waiting/ambush – "*Espera*" (Fig. 3A)

**Figure 3:**
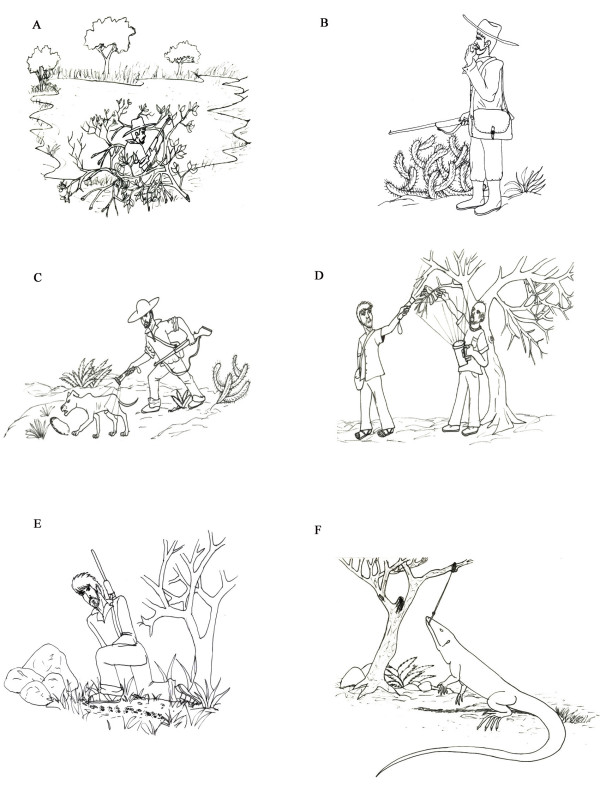
**Hunting techniques used in the semi-arid region of Paraiba State, Brazil**. A – "*Espera*" (waiting/ambush), B – "*Arremedo*" (imitation), C – Hunting with dogs, *D – "Facheado*", E – Tracking, and F – The use of hooks (Illustration: Washington Vieira).

"Waiting" is carried out by hunters, either individually or in pairs, and goes by the local name of "garapa", "bebida", or "pastora". The hunters will first construct a simple hide made from branches near a water hole (some small temporary or permanent reservoir built to capture seasonal run-off). These watering holes are regularly visited by a variety of animals. Additionally, hides can be constructed in places where there are abundant plant food resources such as *Croton sonderianus *(marmeleiro), *Croton *sp. ("velame"), *Jatropha mollissima *(pinhão), or *Cleome spinosa *(muçambê) with seeds or fruits that are eaten by game birds such as *Z. auriculata *and *C. pretiosa, C. picui*, *C. minuta, C. squammata *and *C. talpacoti*.

Once the hide is constructed, the hunter will remain inside, camouflaged among the branches, ready to shoot any animal that approaches. The weapons most commonly used in this technique are shell and mussel-loading rifles.

The animals commonly hunted using this technique are diurnal birds such as *Z. auriculata, C. pretiosa, Columbina picui*, *C. minuta, C. squammata *and *C. talpacoti, N. maculosa *and *L. verreauxi *– although some hunters affirm that they will also shoot other animals (smaller birds and small mammals) that appear and that can be used as food.

This same technique is widely used in other *Caatinga *areas, although it is often adapted to suit the regional vegetation. In areas where the vegetation is more intact and there are large trees and dense forest areas, the hunter may wait in ambush high in the trees.

### Imitation – "*Arremedo*" in the local language (Fig. 3B)

**Figure 4:**
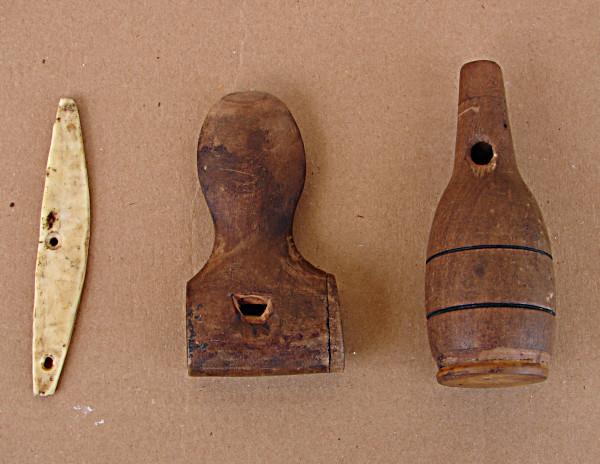
**Whistles used by hunters in the semi-arid region of Paraiba State, Brazil**. The "Arremedo" hunting technique for capturing "mocó" (*Kerodon rupestris*) (left), "lambus" (*Crypturellus tataupa *and *C. parvirostris*) (middle) and "jurutis" (*Leptotila verreauxi*), "ribaçã" (*Zenaida auriculata*), and "asa branca" (*Patagioenas picazuro*) (right).

In this technique, in contrast to "waiting", the hunter doesn't try to ambush the prey at its feeding or drinking sites, but rather attempts to call the prey to within shooting distance by imitating ("arremedar") their songs. This hunting technique is usually practiced individually and is used mainly for game birds. As such, it demands a detailed knowledge of the ecology of the hunted birds, including their reproductive period (their period of "fogo", literally,"fire") and their mating calls. The hunters imitate the bird's call using a whistle ("arremedo") that emits similar sounds (Fig. 4). This instrument can be bought in public markets in the town of Pocinhos or in neighboring cities, although some hunters make their own "arremedos" using plastic or glass materials that come to hand. In some cases the hunter can imitate some of the birds by whistling, without the need of an "arremedo". To use this calling technique, the hunter will walk through the forest until he hears the singing of a bird and he will follow the sound while at the same time trying to call the bird in with the "arremedo".

Besides recognizing the songs of important species, the hunters are able to distinguish the difference between the males the females, since to attract a male the hunter has to imitate the female, and vice versa. As one hunter said: "it is necessary to know if the song is from a male or from a female so we can call in the right animal, so that it thinks that it is its pair and comes to die right next to you...". According to the hunters, during the period of mating ("fogo"), the animals are attracted to the hunters when the opposite sex is imitated ("arremedados").

The animals most hunted using the "arremedo" technique are birds: *C. tataupa, C. parvirostris*, *L. verreauxi*, *C. cristata*, *P. picazuro*, *N. maculosa*, *Z. auriculata *and *Aramides *sp. Some hunters will also imitate and attract small edible mammals such as the rodent *K. rupestris *or mimic the sounds of birds in order to attract foxes and other animals that prey on farm animals such as chickens and guinea-fowl.

### Hunting with dogs (Fig. 3C)

Hunting with dogs can be practiced by one or more men, and generally one or two trained dogs are used. This type of hunting usually takes place at night and the target-prey is usually a mid-sized mammal such as a *C. semistriatus*, *D. novemcinctus*, *E. sexcinctus*, or *T. tetradactyla*.

The hunters usually select areas with intact vegetation (generally mountainous areas) where bigger animals are more abundant and use established game trails. The dogs lead the hunt as they follow the scent of the prey. This form of hunting requires good physical preparation, as long distances can be covered both walking and running. Some hunters, however, prefer to wait in one place while the dogs go off in pursuit of an animal. When the dogs' barking indicates that they have cornered their prey ("acuarem") in a burrow the hunters will follow the barking and attempt to capture the animal by digging it out with shovels and hoes. Some hunters use a kind of iron hook to pull the burrowed ("entocados") animals out. Sometimes the dogs are able to kill the prey during the pursuit and before it can take refuge.

The dogs used for hunting are well trained, most often by the hunter himself as he will have taken them on hunts into the forest while they were still young so that they can learn to hunt with the older and more experienced dogs. Another way to train these dogs is set them to trail and capture armadillos or other wild animals that have been reared at home by the hunters. These wild animals will stimulate the dogs' natural tendency to hunt. Others hunters prefer to buy trained dogs, which are generally sold at high prices (up to US$250). The training and selling of hunting dogs can generate considerable income for some hunters.

During the interviews it became clear that many people hunt because it is emotionally stimulating. It also became apparent that the good hunters, especially those that went after *D. novemcinctus *with dogs, are respected and admired by other hunters as this animal is considered fast and difficult to capture.

Dogs are also be used to hunt *Crypturellus *spp. during the day, but according to the hunters only "perdigueiro" dogs are good trackers. These animals are trained to frighten and flush a "lambu" so that the hunter can shoot it as it takes flight. Once down, the dog will retrieve the bird and bring it back to the hunter.

To train these "lambuzeiro" dogs, the hunters use a fabric ball covered with "lambu" feathers to stimulate the animal's natural predisposition for that kind of hunting. In the municipality of São Mamede, also localized in the semi-arid region, dogs are used to hunt *T. merianae *(tejuaçus) during the day.

### *Facheado *(Fig. 3D)

This hunting technique is usually employed to capture song birds to be kept as pets. In the "facheada" mode, the hunters will go out at night with lanterns to illuminate the birds in their nests. With their vision blurred by the bright light, the startled birds cannot take flight and are easily captured. The principal species captured using this technique are the *I. jamacaii*, *C. brissonii*, *C. yarrellii, S. albogularis*, and *P. dominicana *– birds widely sought after and sold as pets. Additionally, some hunters indicated that *rolinhas *were also captured using this technique, although these birds are usually eaten.

### *Tracking *(Rastreamento) (Fig. 3E)

This technique was used by a only a small number of the hunters who were interviewed (n = 4), and it is mostly directed towards hunting down predators of domestic animals such as felines (*L. tigrinus *and *P. yagouaroundi*) and foxes (*C. thous*), or towards eliminating agricultural pests. Other mammals such as the *E. sexcinctus *and *C. semistriatus*, however, are also tracked by these hunters.

According to the interviewees, when domestic animal such as sheep or chicken are killed, or when a corn plantation is damaged, the small property owners will recruit the hunters to track and to kill or capture the predator animal. The hunters mostly use shotguns in this work.

The tracking hunters have a broad knowledge of these animals' habits and they can readily distinguish their tracks. According to these men, the *L. tigrinus *has nocturnal habits, sleeping during the day in the shade of cactus *Pilosocereus *sp. (facheiro), or others plants in the region, and then going out at night to hunt. *P. yagouaroundi*, on the other hand, has diurnal habits and is primarily responsible for preying on domestic animals. According to the interviewees, when the "gato do mato" kills an animal but buries part of it, it means that it plans to return later to finish its meal. The hunters can often take advantage of this behavior by waiting in ambush near the buried food. The interviewees also stressed that it is necessary to remain down-wind of the prey when tracking so that the animal will not capture the scent of the hunter and take refuge. One interviewee stated that he had killed about two hundred "gatos do mato" during his life. The hunters stated they eat the animals they hunt (e.g. *C. semistriatus, E. sexcinctus, P. yagouaroundi *and *L. tigrinus*) and that some of the animal parts are used for medicinal purposes, as in the case of the fat of *C. semistriatus *or *C. thous*.

### Using hunting traps

Many animal species are captured using traps. The types of traps cited by the hunters included: hooks, "quixó", "arapuca", "arataca". "alçapão", "fojo", "visgo", nooses, or cages for carnivorous or armadillos, which are described as follows.

### Fishing hooks (Fig. 3F)

This type of snare uses a common fishing hook. The hook is attached to some fishing line and then tied to a tree branch so that the hook hangs about 20 cm above the ground. Bait is placed on the hook to attract and snare the prey. This technique is principally used to capture armadillos and "tejuacus".

### *Quixó *(Fig. 5A)

**Figure 5:**
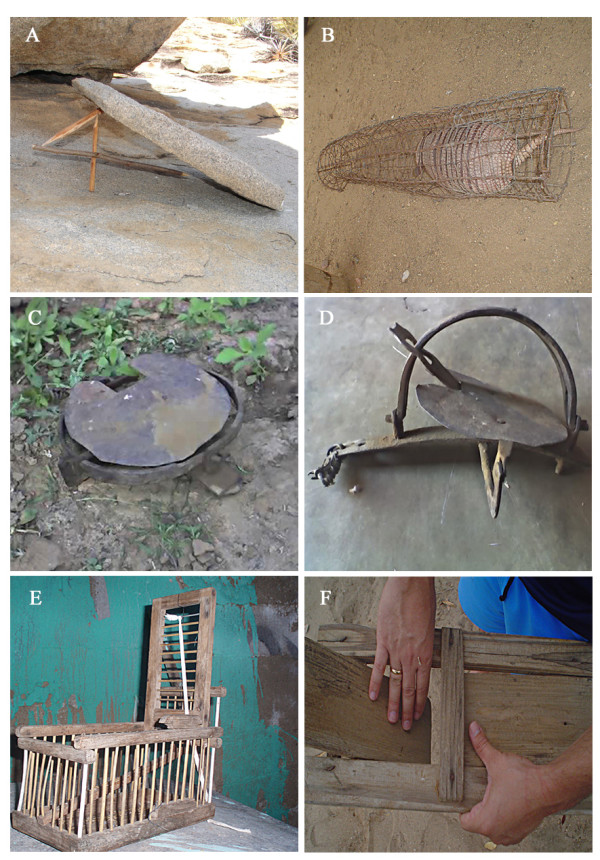
**Hunting traps used in the semi-arid region of Paraiba State, Brazil**. A – "quixó", B – armadillo caught in a "jejeré" or "jequi", C – armed "Arataca", D – desarmed "Arataca", E – "alçapão", and F – "fojo".

The quixó is a fairly simple trap than can be made out of a flat rock (of variable size and shape, depending only on the size of the animal to be captured). The assembled trap is composed of an inclined rock supported by a trigger made of articulated wooden sticks. Fruits or seeds that make up part of the diet of the prey (such as *Ananas *sp. – pineapple, *Croton *sp. – velame, *Jatropha *sp. – pinhão, *Cleome spinosa *– muçambê, or *Croton *– marmeleiro peels) are put under the rock and near the trigger to attract the animal. Any slight touch of the trigger by the animal's movements will cause the "quixó" trap to fall. The animals that are most hunted using this technique are *G. spixii*, *C. aperea *and *K. rupestris*, although other mammals and some birds can also be captured in this way.

### Armadillo cages – "gaiolas" (Fig. 5B)

Cages, also called "jejeré" or "jequi" by some hunters, are commonly used to capture armadillos. This iron trap has a cylindrical shape, with one of the ends being open – but with a triggered door that closes after the animal has entered. This type of trap is used in the cases where the prey "se entoca" (hides in its burrow). The cage is then put in front of the burrow so when the animal comes out looking for food or water it will get caught inside the trap. Often the hunter will simply leave the cage and come back the following day to check to see if the animal has been captured.

The advantage of using this type of trap is that the hunter saves both time and energy in capturing the animal, as he doesn't need to dig it out. Additionally, the hunter can also opt not to kill the animal right away, but rather take it home for fattening ("cevar") – a common course of action, especially with animals like the armadillo.

### "*Arataca*" (Figs. 5C and 5D)

The "arataca" is an iron snap-trap with two jaws that open under pressure. Bait (fruit, chicken heads, eggs, corn, etc) is used to attract the prey, and when the animal steps on the rounded trigger at the base of the trap the two jaws will snap shut on the animal's paw. The animals that are most hunted using this technique are *P. yagouaroundi, L. tigrinus *and *C. thous*, as these animals usually attack domestic animals and destroy plantings. Other animals, like the *C. cristata*, *E. sexcinctus *and *T. merianae *can also be caught in an "arataca", as well as animals without any importance as food resources, such as the *G. vittata *and *D. albiventris*, or even domestic animals (goats, swine, dogs, or domestic cats).

### *Alçapão *(Fig. 5E)

The "alçapão", also called "assaprão" or "gaiola pegadeira", is a type of lightweight birdcage composed of a number of compartments (up to 6). A "campeador" bird (which sings a lot) is placed in the central compartment and the cage is placed in an open field. The songs of the "campeador" bird will attract other male birds that come to "defend" their territory and these will be captured in the other compartments of the cage. Female birds can also be placed in the "assaprão" to attract males. This trap was especially destined to hunt songbirds, which are valued as pets. Another way to use the "assaprão" is to put food inside the cage as bait (primarily "alpiste" seeds).

### - *Fôjo *(Fig. 5F)

The 'fôjo' is a wooden trap made by the hunters, which is used to capture small or medium-sized prey, like *E. sexcinctus*, *G. spixii, C. aperea*" and, less commonly, birds such as *Crypturellus *ssp. The hunters will dig a deep hole and place a large can inside it. At the top of the hole/can they place a suspended lid that will pivot when an animal steps on it, causing the animal to fall into the can. The lid will then quickly pivot back and imprison the animal. To attract the prey, bait (fruits, seeds or eggs – depending on what animal the hunter intends to capture) is placed next to the "fôjo". One advantage of this technique is that a variety of animals can be captured in the same kind of trap. Nevertheless, many animals without food value (in the hunters' opinion) can be captured, such as snakes or "timbus". These animals, besides having little use, can sometimes provoke fatal accidents (as in the case of snakes).

### "*Visgo*"

This method uses an adhesive made (or bought) by the "passarinheiros" (people who specialize in capturing and selling birds) that is applied to a stick – and when a bird lands on the stick it becomes stuck to the glue. According to Gama and Sassi [31], commercially produced "visgo" is not widely used due to its high cost, and it is therefore more common that hunters use a "visgo" prepared from the viscous sap of certain fruit trees *Artocarpus *sp. (Jaqueira) and *Hancornia speciosa *(mangabeira). Often this glue is mixed with the bark of the *Anacardium occidentale *(cajueiro vermelho) to add color and disguise the white tone of the raw sap. The resulting adhesive mass is capable of snaring even large birds. The struggles of birds imprisoned on the "visgo" only worsens their situation as more feathers become ensnared in the glue; and for that reason the trappers have to quickly release the birds or they may die.

### "*Arapuca*" (Fig. 6)

**Figure 6:**
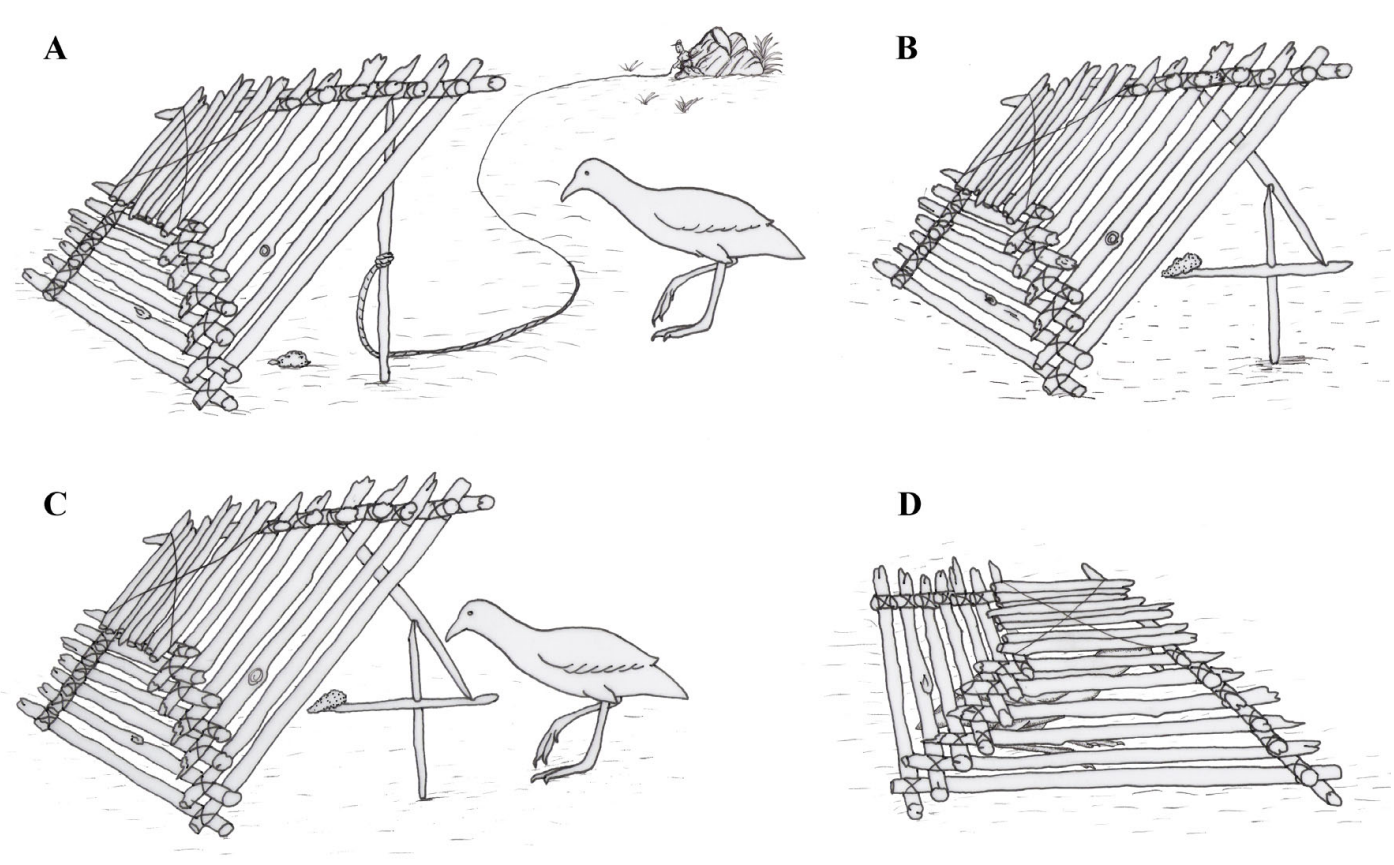
**Arapuca live bird trap ready to be used (A and B), C – Prey attracted by bait and D – Prey imprisoned by Arapuca (Illustration: Washington Vieira)**.

Arapucas (crude cages) are constructed from thin lengths of wood bound by twine or wire into four-sided pyramidal structures (about 40 cm to a side and of equal height). To set the trap, one side of the base of the pyramid is suspended and armed with a trigger mechanism and baited with grains of corn. The hunter will set this trap near a game trail or in place where the animal (usually a bird) will come to feed. When the bird pecks at the corn it will trigger the cage to fall, holding the (usually) live animal until the hunter returns. Depending on the exact size of the "arapuca" it can capture a variable number of individuals and it is commonly used to catch "ribaçãs", "rolinhas", "galinhas dagua", etc.

### Cages for carnivorous (Fig. 7)

**Figure 7:**
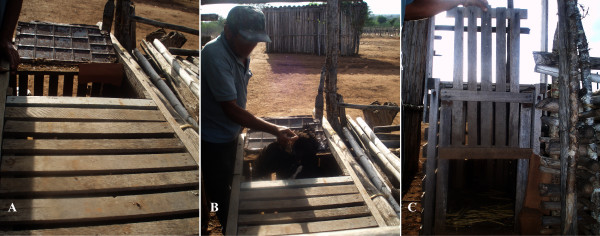
**Partial view of the cages used by hunters to capture carnivorous in the semi-arid region of Paraiba State, Brazil**. A – Upper portion; B – Posterior portion, showing bait (chicken) and C – Anterior portion.

Cages for capturing carnivorous (*L. tigrinus, P. yagouaroundi *and *C. thous*) are called "aratacas" by some hunters. These traps consist of a box made of planks with a door that closes by way of a guillotine mechanism. There is a visible compartment in the back of the trap to hold live bait (generally a chicken). This trap is used by hunters living in rural areas to capture "gatos do mato" and foxes, as these animals usually attack domestic animals and destroy plantings.

### *Nooses *(Fig. 8)

**Figure 8:**
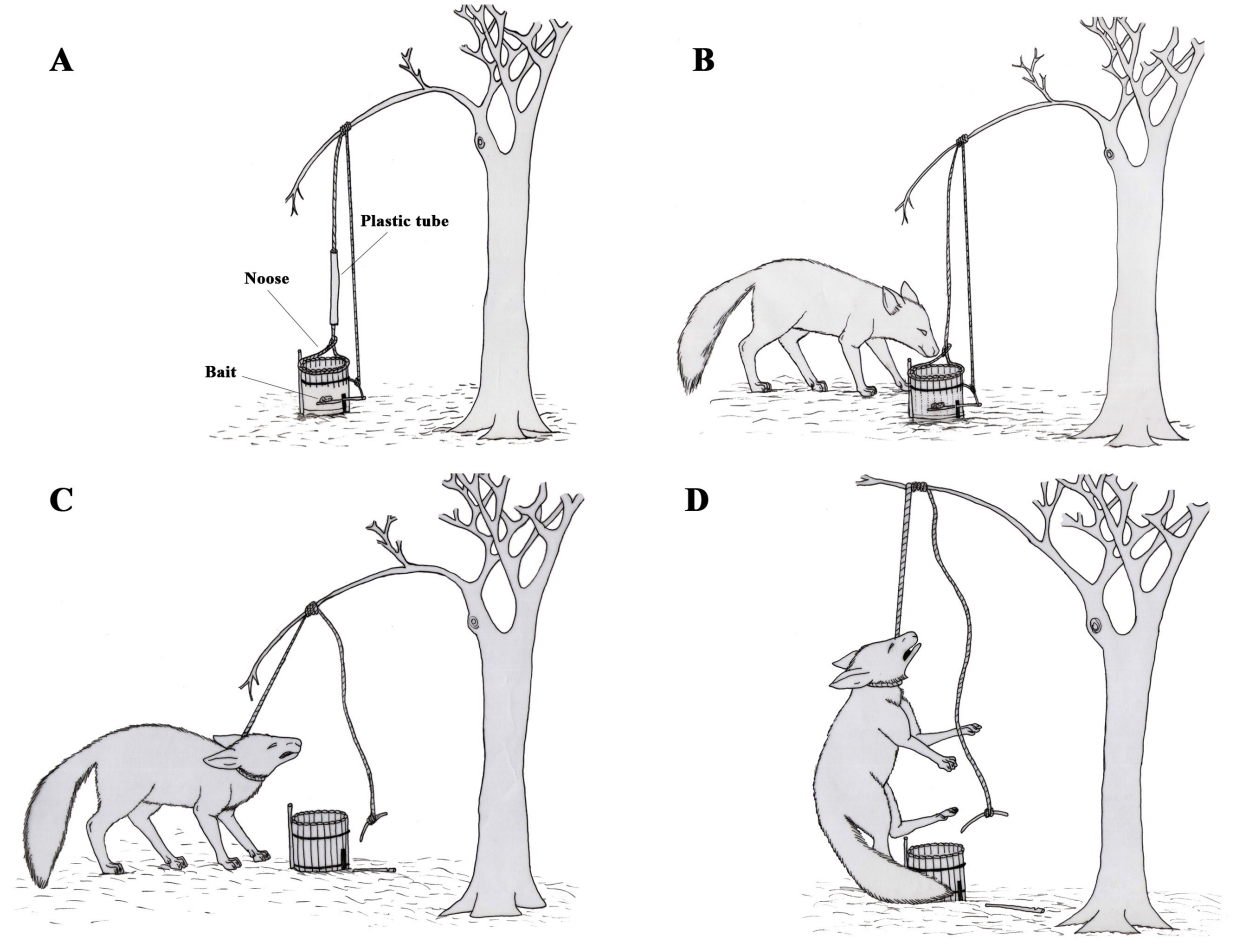
**Noose trap used by hunters in the semi-arid region of Paraiba State, Brazil**. A – Trap prepared for use; B – Prey attracted by bait; C – Prey captured by noose and D – Prey strangled by the noose (Illustration: Washington Vieira).

This type of trap is generally used to capture *L. tigrinus, P. yagouaroundi*, and *C. thous *and consists of a noose made of rope mounted within an open-ended barrel-shaped structure made from lengths of thin wood that is lightly fixed to the ground. There is a small opening near the bottom for the trigger mechanism, which is composed of a thin twig that holds the bait (usually a chicken head or other piece of meat) inside the barrel-like structure that articulates with another twig outside the trap. The second twig is attached to the rope, which is in turn bound to a tensioned branch of a tree; the other end of the rope forms the noose within the trap (see Fig. 8). When an animal puts its head into the trap and grabs the bait, it releases the trigger and the branch snaps upward catching the animal by the neck and choking it or breaking its neck. The rope near the noose will often be covered by a plastic tube to prevent the struggling animal from gnawing through the rope. This trap will often snare animals other than those being actively hunted (e.g. domestic animals).

### Frequency of the use of the techniques and traps described above

**Figure 9:**
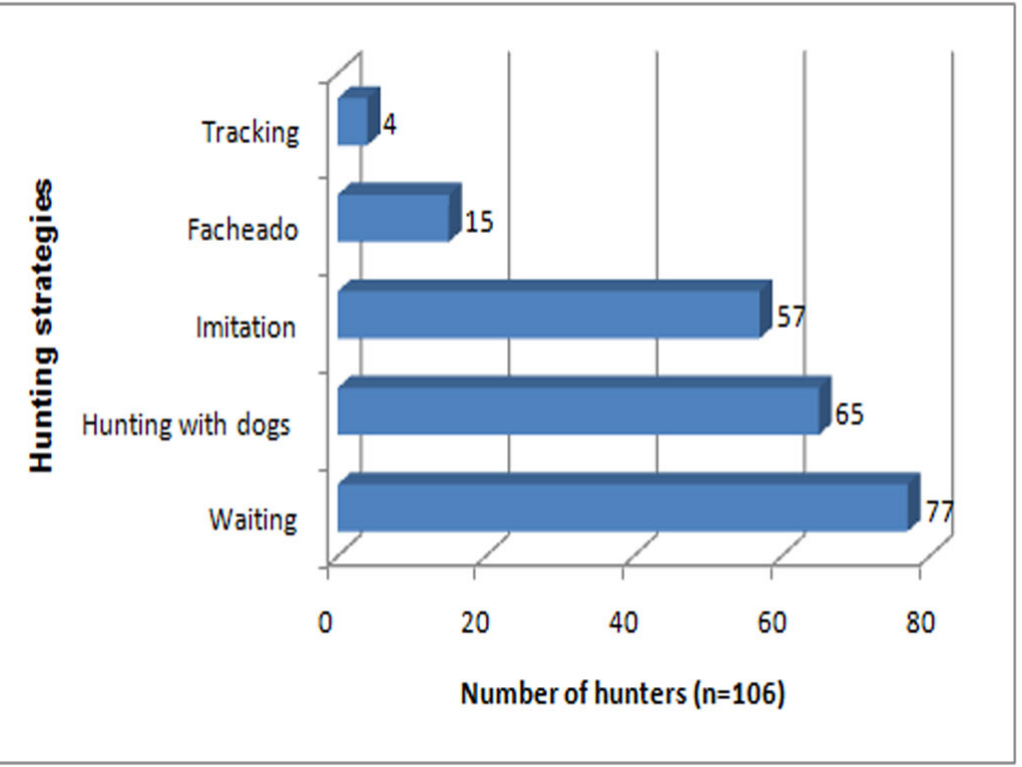
**Use frequency of hunting techniques employed by hunters in the Municipality of Pocinhos, NE Brazil**.

**Figure 10:**
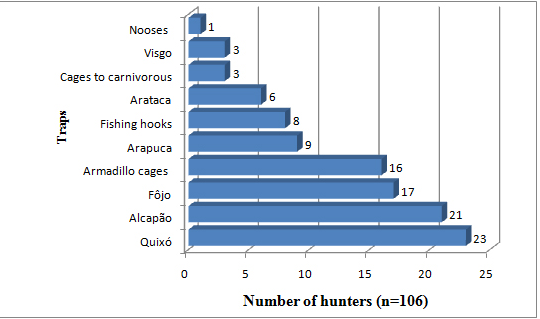
**Use frequency of traps employed by hunters in the Municipality of Pocinhos, NE Brazil**.

The same hunter will use different techniques to catch different animals. Among the techniques available to the interviewees, most (n = 77) simply waited in a hide; 65 hunted with dogs, and 57 called in their prey ("arremedo") (Fig. 9). In terms of traps, the most common was the "quixó" (n = 23), followed by "alçapão" (n = 21), "fojo" (n = 17), and cages for armadillos (n = 16) (Fig. 10)

## Discussion

About 15% of the Brazilian population (more than 25 million people) lives in the dry Caatinga region [32], and the rural population there is characterized by extreme poverty [33]. Due to the adverse environmental conditions in the region, the populations there have developed unique social-environmental structures and a strong dependence on the use of regional natural resources, and they maintain a wide range of interactions with native faunal resources.

Hunting in the study area is practiced by a large number of people and targets a wide variety of species. Vertebrates (birds, reptiles, and mammals) are the principal prey, which is in accordance with other hunting studies undertaken in Brazil and in other parts of the world [11,12,34-43].

The practice of hunting is quite common in the surveyed area, and the people use animal resources in various ways (for medicinal and ornamental purposes, and as food sources or as pets) – which demonstrates the economic and cultural significance of local fauna to people in this region. Capturing songbirds is one of the preferred activities of children and adolescents, and indicates that hunting practices begin in childhood. Besides the utilitarian benefits of hunting, many people (n = 65) admit they hunt for leisure and sport. Our results are in agreement with previous authors who have shown that hunting activities involve socio-economic factors, and that knowledge associated with these practices is transmitted through generations [20,44].

In contrast to any utilitarian value, some species are hunted because they are perceived to represent risks to human health or to domestic stock (e.g. venomous snakes: *Crotalus durissus*, *Micrurus *sp., *Bothrops *sp.) or cause damage to planted areas (e.g. granivorous birds and rodents) or prey on domestic animals (such as the felines). Similar findings were reported from Mato Grosso State, Brazil, where Trinca and Ferrari [20] observed that some local hunters were in favor of the extermination of any predators with the potential to attack humans or their domestic animals, even if these predators lived in their natural forest environment far from human settlements. In relation to snakes, Caatinga inhabitants not only kill the poisonous species, but also non-poisonous individuals (colubrids, boids, and leptotyphlopids) because they provoke fear and repulsion, or because people just consider them as potentially dangerous. The local population also includes amphisbaenians in this context, even though they are not serpents at all, but do have a snake-like body form.

Wildlife-human conflict is a widespread conservation issue of increasing concern to conservationists. Human-wildlife conflicts occur when the needs and behavior of wildlife impact negatively on the goals of humans, or when the goals of humans negatively impact wildlife needs. These conflicts usually occur when wildlife damages crops, injures or kills domestic animals, or threatens or kills people [45]. In truth, whenever human-wildlife conflicts occur both parties lose [46-50] – making human-wildlife interactions a challenging aspect of ecosystem management [51,52].

In this context, the same animal species can represent either a potential resource or a potential economic loss or health risk. *T. merianae*, for example, are predators of domestic birds and their eggs, but this lizard is used as food resource and their fat and their tongues are used as folk remedies [7-10,53,54]. Another example is the rattlesnake (*C. durrisus*), which is a risk to humans and their domestic animals, but the fat from this serpent is widely used in regional medicinal [54-56]. These observations are in agreement with Marques [30], who pointed out that the link between humans and animals is fraught with contradictions and ambiguities, as the native fauna can represent either a resource or a risk to the local people.

As pointed by Ross [57], an important part of the adaptive process of hunting is the co-evolution of hunting technology and general procurement strategies. Our study indicated that a variety of hunting methods are available to exploit the local faunal resources, and individual hunters commonly use more than one technique. This strategy is important because many Caatinga species have a marked seasonality and are abundant mainly during the rainy period, although some are evident all year round. In this context, the possibility of using various hunting techniques permits adaptation to the varying availability/accessibility of game animals.

Shotguns constitute the basic tool of most hunters, even if it is not directly used for killing their prey – for it serves as a potential defense against any unexpected threat during the hunting activities. Previous studies have pointed out that the use of firearms in hunting is almost universal [58]. According to Jerozolimski and Peres [59], weapons like the "cartucheira" shotgun (very common in the present study) are predominant in practically all neotropical regions. Even in the indigenous communities, the use of traditional arms such as the bow and arrow is increasing rare.

Passive hunting (i.e. hunting in which the hunter does not actively search for game) using traditional traps is also relatively frequent. Trap usage has been registered in other regions of Brazil [12,58,60], and in some cases the techniques used are very similar to those employed by the hunters in the present study. Almeida et al. [58], for example, in study carried out in state of Acre, reported the use of a kind of trap called "jequi" that was composed of a basket placed in the entrance of an armadillo den (and similar to the "tatuzeira" used by hunters in present study), and the "mondé", made of a heavy piece of wood set to fall on the animal (which is very similar to the "quixó" used in the present survey area).

The same animal can likewise be hunted using different techniques. The armadillo (*E. sexcinctus*), for example, can be hunted with dogs or captured in "tatuzeiras" traps. In some cases, a combination of techniques will be used, as the hunter may use tracking dogs to locate the armadillo burrow and then set up his trap. The choice of the technique to be used is also related to the ecology of the prey, as the use of dogs is important for terrestrial and nocturnal hunts when the animals are difficult to captured using other techniques. Likewise, the use of whistles (the "arremedo" technique) is important for capturing birds that cannot usually be pursued using dogs.

Knowledge about the habits of game species in an important prerequisite for using traps, as the hunters need to know the correct place to set them up (usually in resting, feeding, or drinking sites). The possibility of encountering a given species is clearly contingent on a combination of factors related to the characteristics of the animals themselves. As such, ecological knowledge and the capacity to interpret tracks and to imitate the animals represent adaptive behavior that allows these men to optimize their hunting success. Previous studies undertaken with hunter/gatherer societies have shown that detailed knowledge about the resources being harvested constitutes a fundamental factor in the success of those hunting or gathering activities [3,61-64].

Some of the techniques used by the hunters in the present research are also practiced in other regions and biomes in Brazil. The "waiting" technique, for example, has been described in the Amazon region – where the hunters will hide in strategic places such as salt-licks ("saleiros" or "barreiros") or fruit trees [12,58,65], or in subsistence plots ("roçados") that are frequently visited by *Agouti paca *(paca) and *Dasyprocta *sp. (cutias) that come to eat *Manihot *sp. (macaxeira) [58,66]. These observations indicate that similar hunting strategies are widespread in different areas and biomes – with local adaptations that depend on the prey and the specific environment.

Another very common hunting strategy in Brazil involves the use of dogs, and it also occurs in the Amazon region [60,65,67]. Diurnal hunts are usually undertaken by a number of hunters (from two to four), and their dogs (two to six) can cover large areas, which increases the probability of capturing some animal. It was observed in the present study that hunting dogs are principally used at night (although diurnal hunts were recorded).

From a conservation perspective, active hunting techniques (waiting, imitation, hunting with dogs, and "facheado") have the greatest impact on the local fauna. The use of firearm and dogs brought greater efficiency to hunting activities. When hunting with dogs, animals are taken regardless of their sex or reproductive state, and pregnant females or those with young are often killed – as can be judged by the fact that many young armadillos were seen being raised by hunters. Additional negative factors associated with hunting with dogs include: 1) although a given species may be sought, other types of animals are often captured or killed, and 2), when a preferred species becomes scarce, other species are hunted in their place. This corroborates the findings of Redford and Robinson [68] who observed that hunters will generally take whatever game they encounter, within their range of acceptable species. Ortiz von Halle [69] also reported that preferences for one or another species disappear when stocks of that favorite species become exhausted, and people will then hunt whatever they can to meet their needs.

Redford and Robinson [68] noted that the use of dogs will usually increase the capture of certain prey species. Observational evidence suggests that dogs primarily aid hunters not by killing the prey, but rather by detecting the animals and flushing them into locations (i.e. burrows, hollow trunks, waterways) where hunters can more easily attack them [70,71]. In an analysis of optimal foraging strategies of Mayangna and Miskito hunters as put forward by Koster [71], it could be seen that both encounter rates and the profitability of prey types can vary dramatically between hunters with dogs and hunters without dogs. Hunters with dogs encountered approximately nine times as many agoutis as unassisted hunters, and nocturnal species such as "pacas" (*Cuniculus paca*) and nine-banded armadillos (*Dasypus novemcinctus*) were typically encountered on day trips only when hunting with dogs [71]. Given these differences, it is reasonable to infer that the species composition of harvests will vary between hunters with dogs and hunters without dogs [72]. Ventocilla et al. (1995) blamed the use of dogs for the local extinction of several wildlife species in rural areas of Panama, and the use of hunting dogs is in fact prohibited in some communities in the Brazilian state of Acre [73,74].

Hunting activities using the "arremedo" technique (which is principally directed towards birds during their reproductive periods) or "facheado" (where the animals are collected directly in their nests) have obvious and direct impacts on animal populations. Another technique that can significantly impact some species is waiting (ambushing). *Z. auriculata*, for example, is a migratory bird that appears in large flocks during the rainy period in the semi-arid region and it can be easily taken by hunters while nesting on the ground. This species has experienced intense hunting pressure on the adult birds and the destruction of its eggs and nests. It can thus be seen that the reproductive period of wild animals often coincides with the principal hunting period for that species, which poses severe challenges for conservation efforts.

Independent of the hunting method used, most hunting activities imply the use of firearms. The adoption of gun hunting, which is far more efficient than traditional methods, almost certainly resulted in a wider range of species being targeted by human hunters (and with greater success). In addition to the proliferation of modern arms, the use of other equipment (such as spotlights) has improved hunting effectiveness and has stimulated commercial and sport hunting in addition to increasing the efficiency of subsistence hunting. Access to technological improvements has allowed hunters to modify their hunting techniques in Brazil and in many other Latin American countries [69,75].

Of the harvested species cited by the hunters above, two are found on Brazil's official list of endangered species: *L. tigrinus *and *C. yarrellii *[76]. Although most of the hunted species are not listed as threatened, over exploitation of the most desired species could lead to local extinctions. Although we were unable to obtain direct measurements of hunting pressure, our interviews with the hunters sought their own evaluations of the impacts of hunting. The great majority of the hunters interviewed (n = 99) stated that the populations of some species appeared to be declining, such as *D. novemcinctus *and *P. yagouaroundi*. Bergallo et al. [77] stated that reduced rates of encounters with particular species are evidence of population declines. As such, we wish to emphasis here the necessity of implementing conservation programs directed towards organizing hunting activities in ways that can guarantee the maintenance of native animal species in the Caatinga region – for the uncontrolled exploitation of wild animal resources not only threatens those species but also the human populations that depend on them.

The fact an extremely threatened species like *L. tigrinus *are perceived as a threat to domestic stock and is actively hunted is of special concern. Hunting has been severely regulated Brazil since 1967 when the first Wildlife Law (N°5197-67) declared that all wildlife species are federally protected and prohibited hunting under any circumstances (except for scientific purposes). A later modification of this law (N° 9605-1998) states that the "destruction" of wild animals is permitted when they are considered "pests" to agriculture or to public health. Permits to eliminate such pests can only be emitted, however, by an unspecified "competent authority."

One of the greatest challenges to wildlife conservation in the Caatinga region is how to integrate human and conservation needs. A large part of the human population in the region lives in extreme poverty [33], and the capture of wild animals is inextricably linked with socioeconomic factors. The persistence of hunting activities in Brazil in spite of the well-known illegality of this practice is closely associated with cultural questions and with the fact that these animals have great nutritional importance to low-income families that cannot obtain sufficient protein resources from domestic animals. As pointed out by Clark [78], poverty is a significant driver of hunting behaviors that place negative pressures on local ecosystems. Leal et al. [21] observed that poverty is considered the principal challenge to the inhabitants of these semi-arid regions, leaving biodiversity conservation with only a small investment priority. The widespread subsistence hunting seen in rural Latin America is an expression of underdevelopment resulting from historical, social, economic and political factors [79].

Although wildlife was mainly used as a protein source in the survey area, hunting cannot be fully explained simply as a need for food. Subsistence hunting in this semi-arid region, as elsewhere, is influenced by a complex array of biological, socio-economic, political, and institutional factors, and understanding this multidimensional context is critical to designing effective conservation solutions. At a local level, the elaboration of management and conservation plans must consider the social and cultural context of the people involved in these activities, and they must be implemented in accord with the populations that use those resources.

An overall reduction in hunting pressure appears to represent the ideal conservation and wildlife management strategy [79], although such reductions may not be feasible in many rural settings for a variety of reasons. In light of these cultural realities, measures designed to guarantee the sustainability of regional hunting by minimizing harvesting impacts on animal populations are fundamental and should include: developing wildlife management educational programs with strong environmental legislation components; the correct enforcement of wildlife laws; and the creation of channels for communication between academic and governmental institutions and human populations involved in hunting. We would also recommend combating sport hunting, an activity that currently attracts large numbers of participants. Limitations on the use of certain hunting technologies, especially firearms, may likewise represent a worthwhile strategy. Additionally, measures that do not directly involve modifying the behavior of the local populations should be considered, such as controlling illegal commercial hunting by outsiders.

Studies on traditional uses of faunistic resources should be carried out with other links to conservation biology and sustainable management of natural resources [80,81]. It must be emphasized, however, that many factors affect animal populations in the Caatinga biome, and the direct consumption of these animals by local populations is only part of the larger problem. According to Leal et al. [21], non-sustainable human activities such as slash and burn agriculture and the continuous use of native pasture for goat and cattle husbandry are causing environmental impoverishment on a huge scale in the Caatinga biome. As such, hunting of wildlife must be considered together with other anthropogenic pressures, such as habitat loss.

## Competing interests

The authors declare that they have no competing interests.

## Authors' contributions

RA, LM, MC, WV and LL – Writing of the manuscript, literature survey and interpretation; RA, LM, MC and WV – Ethnozoological data, and analysis of taxonomic aspects. All authors read and approved the final manuscript.
